# Dysaesthesia in the mental nerve distribution triggered by a foreign body: a case report

**DOI:** 10.1186/1757-1626-2-169

**Published:** 2009-10-28

**Authors:** Panagiotis Kafas, Tahwinder Upile, Nikolaos Angouridakis, Christos Stavrianos, Nikolaos Dabarakis, Waseem Jerjes

**Affiliations:** 1 Department of Oral Surgery and Radiology, School of Dentistry, Aristotle University, Thessalonica, Greece; 2 Head and Neck Centre, University College London Hospital, UK; 3 Department of Surgery, University College London Medical School, UK; 4 Department of ENT, School of Medicine, Aristotle University, Thessalonica, Greece; 5 Department of Endodontics, School of Dentistry, Aristotle University, Thessalonica, Greece; 6 Unit of Oral and Maxillofacial Surgery, UCL Eastman Dental Institute, UK

## Abstract

**Introduction:**

Foreign bodies' entrapments in the mandibular and submandibular regions are quite common.

**Case presentation:**

We report an unusual case of foreign body (amalgam filling) entrapment over the mental foramen causing dysaesthesia in the distribution of the mental nerve. An interesting sign was blue discoloration of the overlaying oral mucosa which was interpreted as amalgam tattooing.

**Conclusion:**

Surgical removal of the foreign object eliminated the reported symptoms.

## Case presentation

A 55-years-old Caucasian female was referred complaining of dull ache and numbness in the lower left mandibular region; the pain radiated towards the midline. No other associated symptoms were reported.

The patient had a remarkable medical history. Dental history included few dental extractions and restorations over the years with no reported complications. No allergies were reported. The patient alcohol consumption was below the recommended safe limit; while she smoked an average of 10 cigarettes/day for the last 20 years.

**Figure 1:**
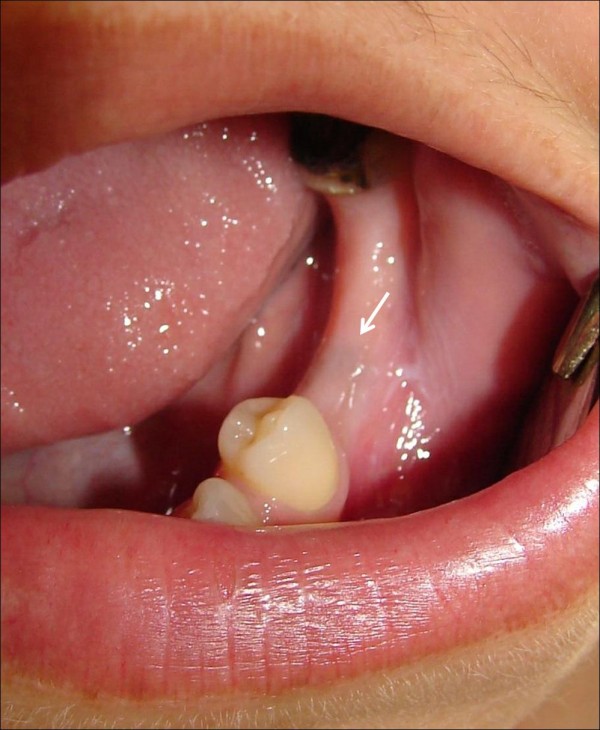
**Intra-oral image showing blue discolouration of the alveolar mucosa (arrow) caused by soft tissue infiltration of amalgam components**.

Intra-oral examination revealed a slight bluish mucosal discoloration distal to the lower left first premolar (Figure 1). The area was tender to palpation; also gentle pressure resulted in pain triggering and radiation to the midline along the body of mandible.

**Figure 2:**
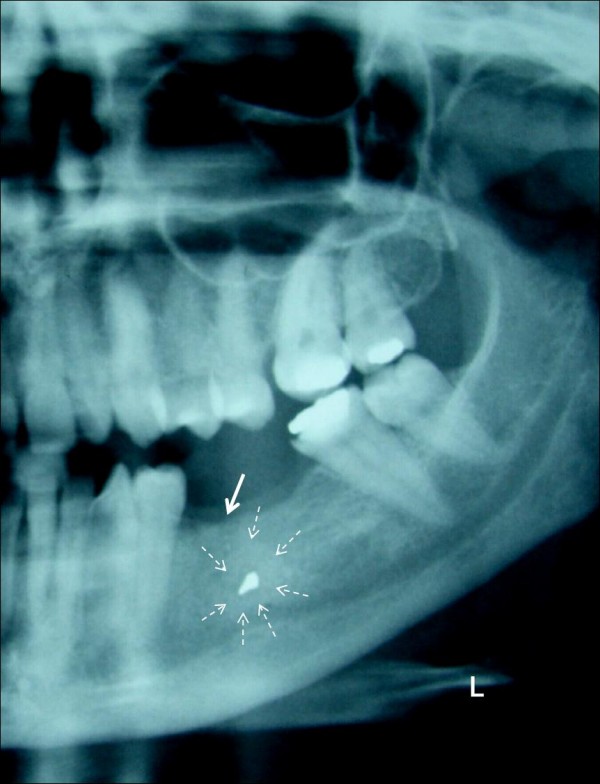
**Unilateral panoramic tomography showing a teardrop-like radio-opaque foreign body in the mandible**. Chronic inflammation and bone remodelling can be seen in the area over the mental nerve and around the foreign body (dashed arrows). Recent dental extraction in the area is apparent on the radiograph as well (non-dashed arrow).

Initial investigations involved a unilateral dental panoramic tomography which revealed a 2-3 mm teardrop-like radiopague area or mass (foreign body) surrounded by or embedded in a radiolucent area (Figure 2). Careful assessment led to the conclusion that this represents an amalgam material that is located over the mental foramen, with surrounding chronic inflammation and bone remodeling. Hence, the pain was triggered by pressure and radiated along the distribution of the mental nerve.

**Figure 3:**
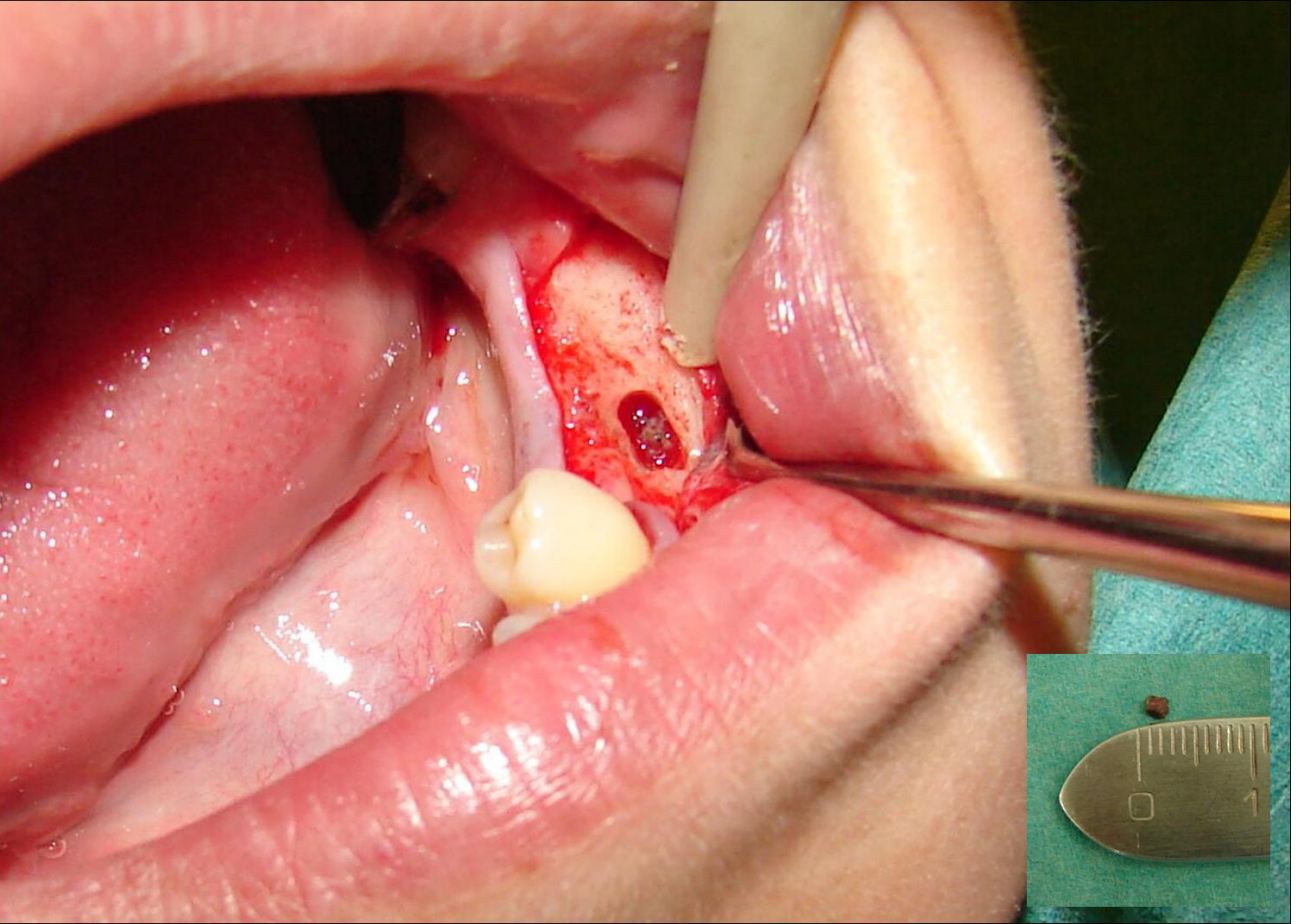
**Removal of the foreign object performed through a minimal-size bony "window" under local anaesthesia**. Inset: the extracted amalgam filling was 2 mm in size.

Open surgical approach under local anaesthesia was employed with care, especially when exposing the mental nerve. Curettage, removal of the foreign body and debridement (Figure 3) took place and the wound was sutured with 3/0 polyglactin. The removed foreign body measured 2 mm in diameter. The patient was covered with NSAIDs and antimicrobials for 1 week. Follow-up at two weeks revealed symptoms resolution. At three months, the patient remained symptom-free.

## Discussion

Foreign bodies' entrapments in the mandibular and submandibular regions are quite common. Inflammation and foreign body granuloma can occur following entrapment of amalgam filling, broken needle, air turbine bur, endodontic filling material, broken toothbrush and sometimes tooth displacement [1-7].

Foreign body (inflammatory) reaction or injury of inferior alveolar nerve (IAN)/mental nerve may be classified into metallic or non-metallic, temporary or permanent, chemico-mechanical or thermal [8]. According to "Steddon classification for peripheral nerve injury", there are three basic injuries: neurapraxia, axonotmesis and neurotmesis [9]. Neurapraxia is a temporary conduction block after mild compression of the nerve trunk (i.e. paraesthesia or dysaesthesia of the lip and chin region in case of IAN/mental nerve) [10]. Axonotmesis, a more serious condition, results from degeneration of the afferent fibers as a result of internal/external irritation (i.e. anaesthesia) [11]. While, in neurotmesis the nerve is completely severed which results in permanent paraesthesia, which can be corrected with microneurosurgical interventions with variable prognosis [9]. In our patient, dysaesthesia (neurapraxia) in the distribution of the mental nerve resulted from compression of the nerve with no structural damage; symptoms can be worsened with chronic irritation and perineural inflammation.

Paraesthesia or dysaesthesia in the distribution of the IAN/mental nerve can be linked to overfilling of root canals close to the nerve [8], severe endodontic infection involving the nerve [12], compression during dental/surgical instrumentation [13,14], mandibular fracture distal to the lingula [15], foreign body reaction [16], pathology [17], orthognathic surgery [18,19] and improper implant placement [15].

Patients presenting with sensory symptoms in the distribution of the IAN/mental nerve area should be assessed carefully. Detailed history, including recent interventions, should be taken; this should be followed by careful examination and radiological investigation. In our patient the presence of amalgam tattooing sign has lead to the conclusion that the problem could be related to amalgam filling. Amalgam tattooing of the oral mucosa results from metallic particles infiltration, which may results in blue discoloration. Investigations was with a unilateral OPG which confirmed the findings.

Surgical removal of the foreign body is the most accepted intervention. In our case, this has resulted in symptoms resolution in a short period of time. However care must be taken during the debridement of the area to avoid any further irritation/damage to the nerve.

In this interesting case, the most accepted justification is that a small amalgam filling (chip) was entrapped in the bony socket of the previously extracted second premolar and caused this local irritation. The host defence mechanism induced inflammatory reaction followed by granulation tissue formation; the chronic inflammation in the area has lead to bone remodeling around the foreign object. It is believed that the origin of the filling material might be from the extracted (restored) tooth or from the 2^nd ^molar which has a missing amalgam filling in its mesial side. The origin of the tattooing of the oral mucosa could be associated with the previously extracted (restored) tooth or occurred soon after entrapment of the amalgam material in the socket.

Clinicians' should be aware of the fact that foreign body's entrapment can lead to neurological complications following dental extractions; and this usually resolves with surgical removal of the insult material. Usually close monitoring is advised in such a case.

## Abbreviations

IAN: Inferior Alveolar Nerve.

## Competing interests

The authors declare that they have no competing interests.

## Authors' contributions

PK, TU, NA, CS, ND, WJ were major contributors in assessing the case data, reviewing and writing the manuscript. All authors read and approved the final manuscript.

## Consent

Written informed consent was obtained from the patient for publication of this case report and accompanying images. A copy of the written consent is available for review by the Editor-in-Chief of this journal.
